# Radial pulse harmonic analysis for cardiometabolic assessment: a computational and translational mini review

**DOI:** 10.3389/fendo.2026.1867056

**Published:** 2026-07-03

**Authors:** Chung-Hung Wu, Jian-Jung Chen, Hsientsai Wu

**Affiliations:** 1 Department of Chinese Medicine, Taichung Tzu Chi Hospital, Buddhist Tzu Chi Medical Foundation, Taichung, Taiwan; 2 School of Post-Baccalaureate Chinese Medicine, Tzu Chi University, Hualien, Taiwan; 3 Graduate Institute of Acupuncture Science, China Medical University, Taichung, Taiwan; 4 Institute of Medical Sciences, Tzu Chi University, Hualien, Taiwan; 5 Department of Electrical Engineering, Dong Hwa University, Hualien, Taiwan

**Keywords:** cardiometabolic assessment, computational cardiovascular physiology, harmonic analysis, pulse waveform analysis, radial pressure pulse (RPP), vascular hemodynamics

## Abstract

**Purpose:**

Radial pulse harmonic analysis has emerged as a quantitative frequency-domain approach for non-invasive assessment of cardiovascular and metabolic physiology through characterization of arterial hemodynamics. By decomposing radial pressure pulse waveforms into harmonic components, this method provides physiologically interpretable information regarding arterial compliance, wave reflection, vascular resistance, and ventricular–arterial coupling that may not be fully captured by conventional blood pressure measurements or time-domain indices.

**Methods:**

This mini review summarizes the theoretical foundations, signal-processing framework, methodological developments, and clinical applications of radial pulse harmonic analysis. Particular emphasis is placed on its role in the assessment of hypertension, diabetes mellitus, coronary artery disease, vascular aging, and autonomic dysfunction, as well as its integration with emerging computational cardiovascular technologies.

**Results:**

The reviewed evidence demonstrates that harmonic parameters are consistently associated with arterial stiffness, endothelial dysfunction, autonomic dysregulation, vascular remodeling, and age-related hemodynamic alterations. Advances in sensor technology, signal acquisition protocols, signal-processing algorithms, and computational modeling have improved the reproducibility and translational potential of harmonic analysis. In addition to established cardiometabolic applications, emerging approaches such as Reservoir-Excess Pressure Analysis, photoplethysmography-derived biomarkers, wearable sensing technologies, and computational fluid dynamics-based vascular modeling may further enhance the physiological interpretation and clinical utility of pulse waveform analysis. Collectively, these developments support the use of harmonic analysis as a complementary biomarker framework for non-invasive cardiovascular assessment.

**Conclusions:**

Radial pulse harmonic analysis represents a promising computational and translational framework for non-invasive vascular assessment and cardiometabolic risk evaluation. By providing quantitative insights into vascular function and systemic hemodynamics, harmonic analysis may complement existing cardiovascular biomarkers and contribute to precision cardiovascular medicine. Nevertheless, further methodological standardization, mechanistic validation, and large-scale prospective studies are required before broader clinical implementation can be established.

## 1 Introduction

The radial artery represents a practical and physiologically relevant site for non-invasive arterial waveform acquisition because of its superficial anatomical location, stable vascular geometry, and established coupling with central hemodynamic changes. Advances in biomedical signal processing and computational cardiovascular physiology have enabled radial pressure pulse (RPP) waveforms to be quantitatively analyzed using frequency-domain approaches, thereby providing physiologically interpretable information regarding arterial compliance, wave reflection, vascular resistance, and autonomic modulation (1, 2).

From a cardiovascular physiology perspective, the arterial pulse is generated by left ventricular ejection and subsequently modified by arterial compliance, vascular impedance, peripheral resistance, and wave reflection throughout the arterial tree (3). Consequently, pulse waveforms contain rich hemodynamic information that extends beyond conventional systolic and diastolic blood pressure measurements. The radial artery provides a convenient and reproducible site for capturing these physiological signals, making it attractive for non-invasive cardiovascular assessment and longitudinal monitoring (4).

The arterial pulse is a propagating pressure waveform generated by left ventricular ejection and shaped by the integrated effects of arterial compliance, vascular impedance, peripheral resistance, and wave reflections throughout the arterial tree (5). The radial artery, the classical palpation site in TCM, reflects peripheral manifestations of central hemodynamics and contains information on both central and local vascular properties. Therefore, converting qualitative pulse descriptions into quantitative hemodynamic and biomechanical parameters provides a natural translational bridge between traditional pulse diagnosis and modern clinical medicine (5, 6). Early attempts to objectify pulse diagnosis primarily focused on time-domain waveform parameters, such as pulse amplitude, rise time, cycle interval, and dicrotic notch features; although informative, these metrics provide limited descriptions of a complex oscillatory signal (7). The arterial pulse is intrinsically quasi-periodic and composed of multiple frequency components, each reflecting different aspects of cardiac mechanics and vascular properties. Accordingly, frequency-domain and harmonic analyses, which are techniques that decompose the pulse waveform into a fundamental frequency and higher-order harmonics, have emerged as powerful tools for extracting physiologically meaningful features from radial pulse signals (8–10). Harmonic analysis, based on Fourier decomposition, yields the energy distribution across harmonics, reflecting both the mechanical properties of the arterial system and the interaction between forward-propagating and reflected waves (9). In general, lower-order harmonics are more closely related to cardiac output and central arterial compliance, whereas higher-order harmonics are increasingly influenced by peripheral resistance, microvascular function, and arterial stiffness (10, 11). Accumulating clinical and experimental evidence indicates that hypertension, diabetes mellitus, coronary artery disease, and age-related arteriosclerosis significantly alter the harmonic structure and spectral energy distribution of arterial pulse waves (11–14). From the perspective of TCM scientification, harmonic analysis offers the distinct advantages of being both mathematically rigorous and physiologically interpretable, enabling the transformation of qualitative pulse descriptors into reproducible quantitative biomarkers. Several studies have reported associations between classical pulse qualities and spectral features. Several studies have suggested that variations in harmonic energy distribution and waveform morphology may reflect underlying differences in vascular compliance, wave reflection, and peripheral hemodynamics. Nevertheless, the physiological interpretation of specific harmonic patterns remains an active area of investigation, and further mechanistic studies are needed to establish standardized clinical applications (15, 16). Although these correspondences remain preliminary, they provide a plausible mechanistic link between traditional pulse theory and modern vascular physiology. Technological advances have further enhanced clinical feasibility.

High-resolution pressure sensors, applanation tonometry devices, and photoplethysmography (PPG)-based systems now enable non-invasive acquisition of radial pulse waveforms in both outpatient and bedside environments (17, 18). When combined with standardized preprocessing procedures, including baseline-drift correction, noise filtering, cycle alignment, and normalization, harmonic indices exhibit substantially improved reproducibility and reliability across clinical studies (7, 18). Clinically, harmonic parameters have demonstrated sensitivity to diverse cardiovascular and metabolic disorders. For instance, patients with hypersensitivity frequently exhibit enhanced high-order harmonic components or altered harmonic decay rates, changes that correlate with increased arterial stiffness and elevated central blood pressure (11, 19). In type 2 diabetes mellitus (T2DM), microvascular dysfunction and autonomic neuropathy are associated with distinct alterations in harmonic indices (12). Age-related vascular remodeling commonly manifests as a redistribution of spectral energy toward lower frequencies, consistent with reduced arterial compliance and increased pulse wave velocity (13, 20). Collectively, these findings support the potential of pulse harmonic indices as non-invasive vascular biomarkers. Nevertheless, several important challenges remain. Substantial heterogeneity in sensor technologies, measurement protocols, preprocessing pipelines, and definitions of harmonic indices complicates cross-study comparisons and hinders robust meta-analyses and clinical standardization (21). Moreover, several investigations linking TCM pulse categories to harmonic measures rely on small or single-center cohorts, limiting the generalizability of their findings (22). Finally, although harmonic analysis often reveals statistically significant differences between disease and control groups, the incremental diagnostic value of harmonic indices beyond established clinical metrics, such as pulse wave velocity, central blood pressure, and heart rate variability, requires systematic evaluation (23, 24).

Accordingly, this Mini Review aims to: (i) summarize the physiological foundations of radial pulse harmonic analysis; (ii) review current methodologies for signal acquisition, preprocessing, and harmonic extraction; (iii) examine clinical applications in cardiometabolic and vascular disorders; (iv) discuss emerging computational cardiovascular approaches, including Reservoir-Excess Pressure Analysis, photoplethysmography-derived biomarkers, and computational fluid dynamics; and (v) identify current limitations and future directions for the development of harmonic analysis as a translational tool for non-invasive cardiovascular assessment.

## 2 Physiological basis of radial pulse waveforms

### 2.1 Search strategy and selection criteria

This review was conducted in accordance with the Preferred Reporting Items for Systematic Reviews and Meta‐Analyses (PRISMA 2020) guidelines (25). A systematic literature search was performed in PubMed, Web of Science, Scopus, and Embase databases covering the period between January 2000 and May 2026. The search strategy combined the following keywords using Boolean operators: radial pulse waveform, arterial pressure wave, harmonic analysis, Fourier analysis, arterial compliance, pulse wave velocity, wave reflection, and traditional Chinese medicine pulse diagnosis.

Eligible studies were required to: (1) involve human participants or clinically relevant experimental models; (2) investigate the physiological mechanisms of arterial pressure waves, harmonic analysis, or radial pulse measurements; and (3) provide quantitative and reproducible analytical methods. Studies were excluded if they were duplicates, non-English articles, conference abstracts without full text, or reports with insufficient methodological detail. After screening titles, abstracts, and full texts, a total of 35 studies were included for qualitative synthesis.

### 2.2 Generation of arterial pressure waves: cardiac output, ejection velocity, and pulse wave velocity

Arterial pressure waves originate from the left ventricular ejection of blood into the aorta during systole. The cardiac output and left ventricular ejection velocity determine the initial amplitude and upstroke of the pressure wave (26, 27). Once generated, the pressure wave propagates along the arterial tree toward the peripheral circulation, and the speed of propagation is defined as the pulse wave velocity (PWV), which is primarily determined by arterial wall elasticity and geometry (26, 28).

In young and healthy arteries, PWV is relatively low; however, vascular aging and arteriosclerosis significantly increase PWV due to enhanced arterial stiffness (29, 30). The arterial system behaves as a distributed impedance network, where the pulsatile pressure signal undergoes continuous transmission and reflection (27, 31). Consequently, the radial pulse waveform represents a superposition of forward-traveling and reflected waves, simultaneously encoding cardiac pumping dynamics and global arterial mechanical properties.

### 2.3 Harmonic analysis of radial pulse waveforms: methodology and physiological interpretation

#### 2.3.1 Signal decomposition of radial pressure pulse using fourier harmonic analysis

The HA of RPP in the current study comprised two processes: (1) the peak alignment process and (2) the harmonic component’s computation.

A. Peak Alignment Process

RPPs were adopted for harmonic component computation after the peak alignment process. Ensemble averaging was performed using multiple consecutive cardiac cycles (typically 5–8 cycles), consistent with previous harmonic analysis studies. Briefly, RPP signals were continuously acquired over a 6-second recording period and segmented into individual cardiac cycles based on systolic peak detection. Following peak alignment, consecutive pulse cycles were temporally synchronized and ensemble-averaged to generate a representative waveform with reduced noise and beat-to-beat variability. The resulting averaged waveform was subsequently used for Fourier-based harmonic decomposition and frequency-domain analysis. In this case, {y[n]} is a sampled period’s discrete signal, and therefore, {y[n]} = {y[n + k × 3000]}, k = 0,1,2,…,7. These sampled data can be decomposed as follows:

(1) $$\left\{\text{y}\left[n\right]\right\}=\frac{1}{3000}\overset{3000-1}{\underset{\text{k}=0}{\sum }}Y\left(\text{k}\right)e^{i\frac{2\pi }{3000}\text{kn}},\text{ n}=1,\;2,\;\ldots ,18000,$$

where {Y(k), k = 0, 1, 2, …,10} is the first 11 harmonic components of y[n], as suggested in (29). It can also be represented as follows:

(2) $$\left\{\text{Y}\left(\text{k}\right)\right\}=\overset{3000-1}{\underset{\text{n}=0}{\sum }}\text{y}\left[\text{n}\right]e^{-i\frac{2\pi }{3000}\text{kn}},\text{ k}=0,1,2,\ldots ,10.$$

**Figure 1:**
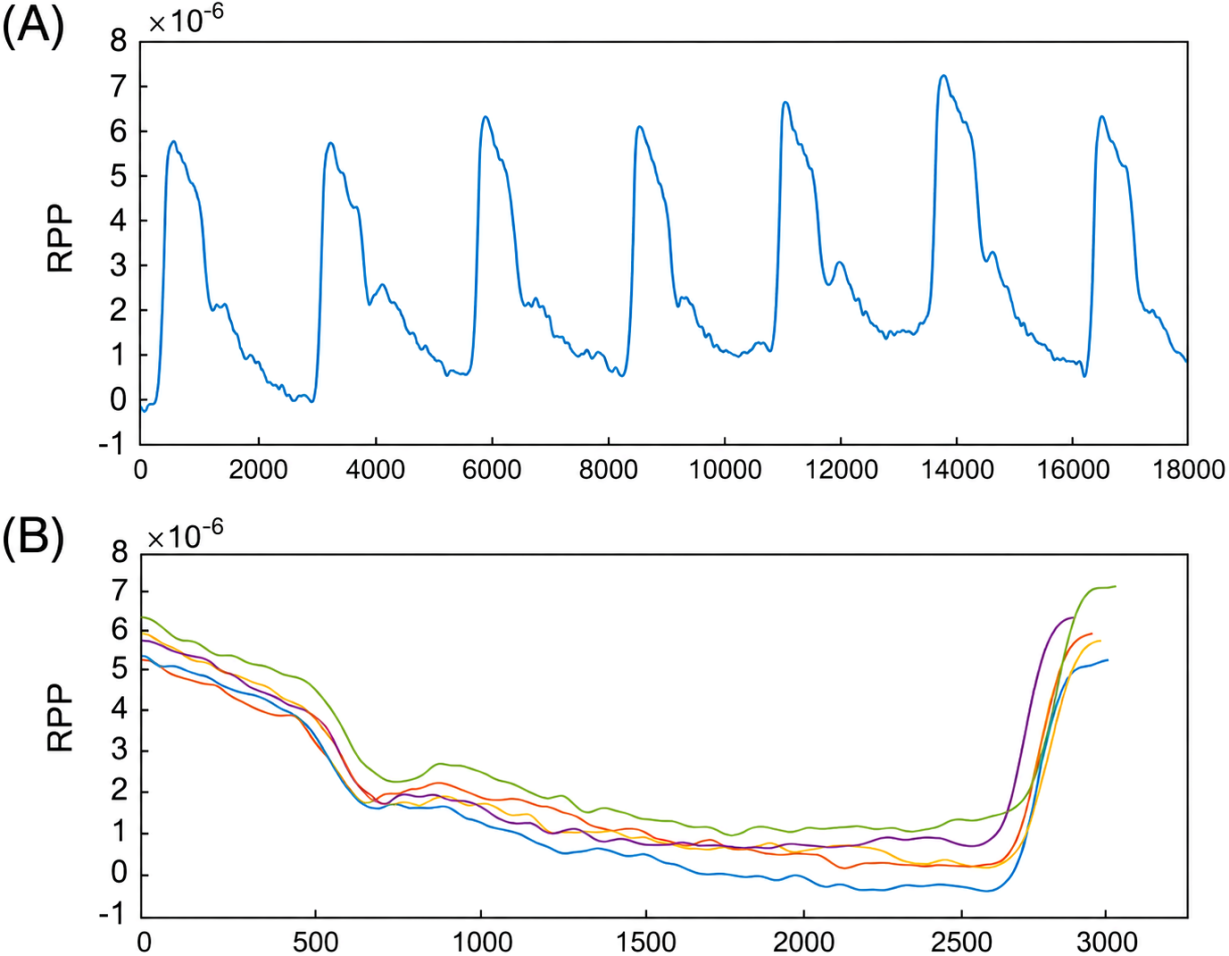
Radial pressure pulse acquisition and peak alignment procedure. **(A)** Representative radial pressure pulse (RPP) signals non-invasively acquired from the radial artery using a pressure sensor. Continuous pulse waveforms were digitized and recorded under standardized resting conditions for subsequent signal processing and frequency-domain analysis. **(B)** Peak alignment of consecutive pulse cycles. Individual cardiac cycles were identified according to their systolic peaks and temporally aligned to minimize cycle-to-cycle variability. The aligned waveforms provide a consistent basis for ensemble averaging and subsequent Fourier-based harmonic decomposition. Harmonic Component Computation.

Due to the RPP being a quasiperiodic signal, all values of Ti (T1, T2,…,Tnp) are similar, but not identical, in Equations 1 and 2. All RPP signals were separated sequentially via the process of peak alignment. Therefore, the total np periods of the RPP could be determined precisely using the ensemble averaging process after peak localization, as shown in **Figure 1**.

All RPP signals were separated sequentially by the np peaks after the peak alignment process. Additionally, the computation of the harmonic component progressed as follows:

Step 1. The Fourier series coefficients for each period of the RPP were found in Equation 3:

(3) $$\left\{\text{Y}\left(\text{k},\text{j}\right)\right\}=\overset{3000-1}{\underset{\text{n}=0}{\sum }}y_{\text{j}}\left[\text{n}\right]e^{-i\frac{2\pi }{3000}\text{kn}},\text{ k}=0,1,2,\ldots ,10$$

where yj[n] indicates the jth-period pulse and k indicates the kth harmonic component of the np period of the RPP.

Step 2. The average amplitude value of each period of the RPP was calculated:

(4) $$\left|Y\left(\text{k},\text{ j}\right)\right|=\frac{1}{3000}\underset{\text{j}}{\sum }\left|{\text{y}}_{\text{j}}\left[\text{n}\right]\right|,\text{ j}=1,2,\ldots ,n\text{p}.$$

The mean of the j vectors of {Y(k,j)} is a vector with j means in Equation 4.

Step 3. The coefficients of the Fourier series for each period were normalized into the format ({Y(k,j)})/|Y(k,j)|, j = 1,2,3,…,np. The kth row of period j of {Y(k, j)} was divided by the absolute mean of {Y(k,j)} of the jth period.

Step 4. The representative coefficients of the harmonic component were found as follows:

(5) $${\text{C}}_{\text{k}}=\frac{1}{n\text{p}}\underset{\text{j}}{\sum }\frac{\left\{Y\left(\text{k},\text{ j}\right)\right\}}{\left|Y\left(\text{k},\text{ j}\right)\right|},\text{ k}=0,1,2,\ldots ,10;\text{ j}=1,2,\ldots ,n\text{p}.$$

C_0_ in Equation 5 is defined as baseline/DC harmonic component of the averaged pulse waveforms for the RPP signals. The other coefficients of the harmonic component [i.e., C_1_–C_10_ in Equation 5] reflect the harmonic components between the heart rate and the harmonic frequencies of the arterial system (7, 32). Finally, as listed in Step 4, the normalized Fourier coefficient was determined as the mean of the normalized harmonic components calculated over the RPP periods included in Equation 5. The MATLAB scripts developed for radial pulse peak alignment and harmonic analysis in this study are included as **Supplementary Materials** at the end of the manuscript.

**Figure 2:**
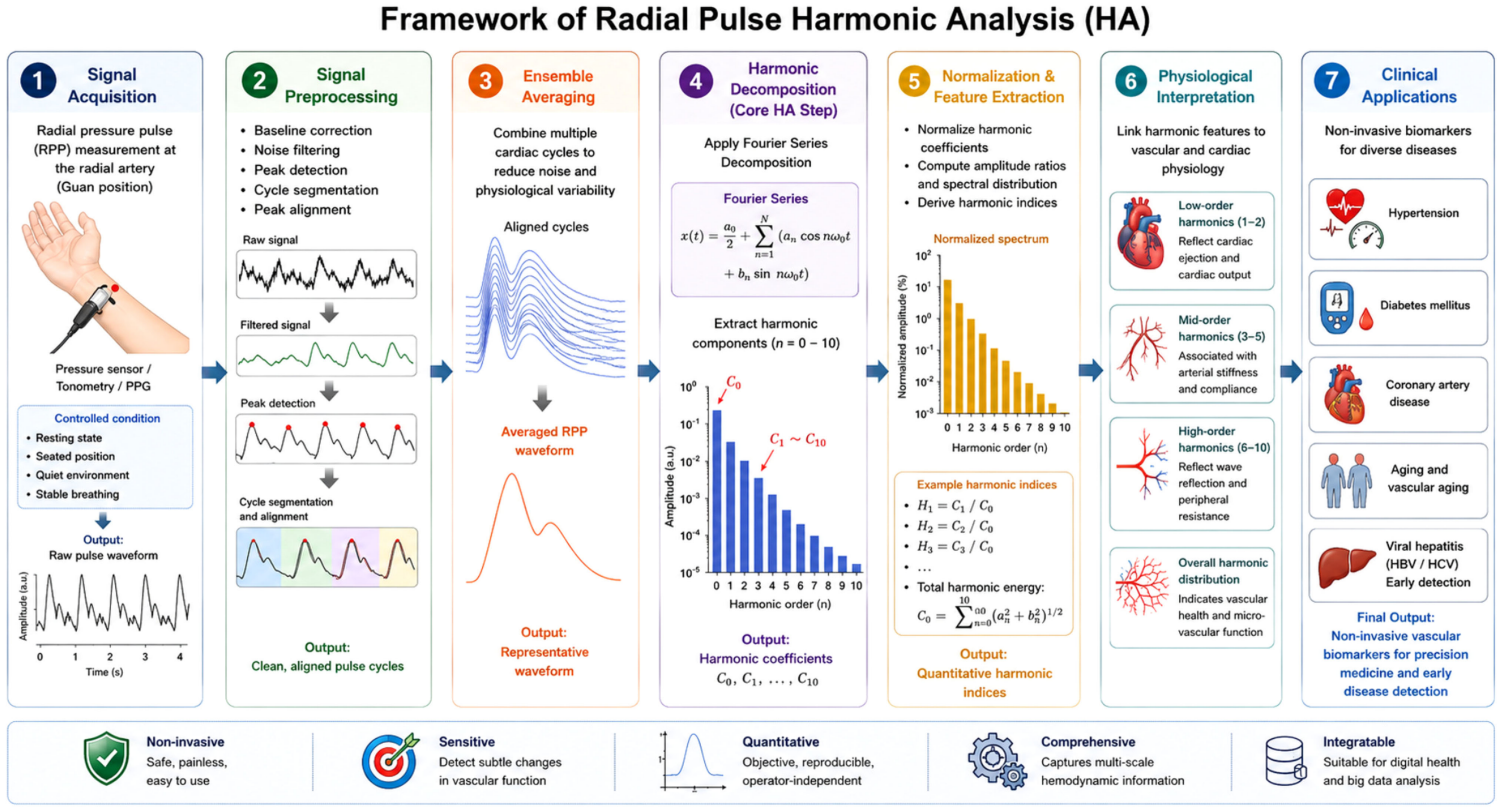
Framework of radial pulse harmonic analysis (HA).

In summary, RPP signals were non-invasively acquired from the radial artery using a computerized pulse waveform acquisition system equipped with a high-fidelity pressure sensor. Participants were examined in a seated and relaxed posture following an adequate resting period to minimize autonomic and hemodynamic fluctuations during signal acquisition. The sensor was positioned perpendicular to the skin surface to ensure stable contact pressure and optimal waveform quality. Continuous pulse signals were recorded for multiple cardiac cycles and digitized at a fixed sampling frequency for further analysis (**Figure 2**). Before spectral analysis, raw RPP signals underwent preprocessing, including baseline correction, noise filtering, and peak alignment to ensure cycle-to-cycle consistency. Several consecutive pulse cycles were ensemble-averaged to generate a representative pulse waveform for each participant, thereby reducing the influence of transient noise and respiration-related variability. The averaged waveform was then decomposed using Fourier series expansion to obtain harmonic components from the zeroth (C_0_) to the tenth (C_10_) order. Each harmonic coefficient quantified the amplitude contribution of a specific frequency component to the overall pulse waveform (**Figure 2**). RPP signals were noninvasively acquired at the radial artery (Guan position) using a high-fidelity pressure sensor under standardized resting conditions. The signals were digitized at a fixed sampling frequency and preprocessed through baseline correction, noise filtering, and peak detection. A peak alignment procedure was applied to segment consecutive pulse cycles, ensuring temporal consistency across quasi-periodic waveforms, as illustrated in **Figure 2**. Following peak alignment, multiple pulse cycles were ensemble-averaged to generate a representative waveform, thereby reducing the influence of noise and inter-cycle variability. The averaged waveform was subsequently decomposed using Fourier series expansion. For each cardiac cycle, Fourier coefficients were computed according to Equations 3-5, and harmonic components from the zeroth to the tenth order (C_0_–C_10_) were obtained. To facilitate inter-subject comparison, harmonic coefficients were normalized based on their corresponding mean amplitudes. The normalized harmonic indices represent the relative spectral energy distribution of the pulse waveform and were calculated as the average of harmonic components across all aligned cycles. As summarized in **Figure 2**, this analytical pipeline enables the transformation of RPP signals from time-domain waveforms into frequency-domain representations. These indices quantitatively characterize the spectral properties of the arterial pulse and reflect underlying hemodynamic mechanisms, including cardiac output, arterial compliance, wave reflection, and peripheral resistance. The derived harmonic parameters were subsequently used for statistical analysis and clinical interpretation.

#### 2.3.2 Physiological determinants of harmonic energy distribution: compliance and wave reflection

Wave reflections occur at arterial bifurcations and peripheral resistance sites, causing the interaction of forward and reflected pressure waves (33). Using Fourier-based harmonic analysis, the radial pulse waveform can be decomposed into a fundamental frequency and higher-order harmonics, yielding a complete spectral energy profile (12, 19, 32).

The fundamental harmonic primarily reflects cardiac output and central arterial compliance, whereas higher-order harmonics are increasingly influenced by peripheral resistance, microvascular compliance, and wave reflection (19, 32, 33). Clinical studies demonstrate that hypertension, diabetes mellitus, and aging significantly alter harmonic energy distributions and harmonic decay patterns (12, 19, 32).

In arterial stiffening, reflected waves return earlier during systole, augmenting central pressure and increasing high-order harmonic energy (19, 34). Harmonic distortion indices have been proposed as surrogate markers of arterial stiffness and correlate closely with PWV and central systolic pressure (9). In patients with type 2 diabetes mellitus, microvascular dysfunction and autonomic neuropathy lead to characteristic harmonic alterations (35).

These findings confirm that harmonic energy distribution provides a sensitive quantitative linkage between ventricular ejection, arterial compliance, wave reflection, and vascular remodelling, establishing a strong physiological foundation for pulse harmonic analysis.

### 2.4 Rationale for radial artery-based pulse waveform assessment

The radial artery represents a practical and physiologically relevant site for non-invasive pulse waveform acquisition because of its superficial anatomical location, stable vascular geometry, and relatively low susceptibility to motion artifacts. These characteristics facilitate reliable signal acquisition in both clinical and research settings.

From a hemodynamic perspective, the radial artery is located along the central-to-peripheral arterial transmission pathway and exhibits a strong physiological relationship with central arterial pressure dynamics. Previous studies have demonstrated that radial artery waveforms retain substantial information regarding ventricular ejection, arterial compliance, vascular impedance, and wave reflection phenomena. Furthermore, generalized transfer function approaches have enabled the reconstruction of central aortic pressure waveforms from radial artery measurements with acceptable accuracy, supporting the physiological relevance of peripheral pulse signals for cardiovascular assessment (36, 37).

Recent advances in pulse waveform acquisition technologies, including applanation tonometry, high-resolution pressure sensors, PPG, and wearable sensing platforms, have further enhanced the feasibility of non-invasive radial pulse monitoring (17, 19). When combined with standardized preprocessing procedures, such as baseline correction, noise filtering, cycle alignment, ensemble averaging, and normalization, harmonic indices demonstrate improved reproducibility and inter-study consistency (7, 18, 38).

In addition, integration of harmonic analysis with complementary hemodynamic parameters, including PWV, arterial input impedance, wave reflection indices, and Reservoir-Excess Pressure Analysis, may provide a more comprehensive assessment of vascular function and cardiovascular risk (39). These multimodal approaches enable simultaneous characterization of arterial compliance, peripheral resistance, vascular load, and systemic hemodynamic regulation.

Taken together, the radial artery provides a scientifically justified and clinically robust platform for non-invasive pulse waveform analysis. Its anatomical accessibility, physiological relevance, and compatibility with modern signal-processing technologies support its continued development as a quantitative tool for cardiovascular and cardiometabolic assessment.

## 3 Clinical applications of radial pulse harmonics

Radial pulse harmonic analysis has been widely used in clinical research as a non-invasive tool for assessing cardiovascular mechanics, microvascular function, and systemic vascular regulation. By decomposing the arterial pulse waveform into its frequency-domain components, this method yields quantitative indices that reflect the complex interaction among cardiac ejection, arterial compliance, wave reflection, and peripheral resistance (40, 41). To facilitate readers’ understanding of the physiological and analytical meaning of radial pressure pulse under Fourier harmonic analysis, this section synthesises the present clinical evidence supporting the application of radial pulse harmonics in major chronic disorders, particularly emphasising viral hepatitis screening, hypertension, diabetes mellitus, coronary artery disease, and vascular aging.

### 3.1 Exploratory perspectives on systemic physiological applications of radial pulse harmonic analysis

Recent advances in pulse waveform analysis have suggested that radial pulse harmonic analysis may be sensitive to subtle systemic physiological alterations beyond conventional cardiovascular disorders. In particular, growing evidence indicates that chronic inflammation, autonomic dysregulation, endothelial dysfunction, and vascular remodeling can influence the spectral distribution of arterial pulse waveforms, thereby altering harmonic characteristics detectable through frequency-domain analysis. Several exploratory studies have suggested that harmonic redistribution patterns may reflect systemic vascular responses associated with metabolic, inflammatory, and infectious conditions. These observations are physiologically plausible because chronic inflammatory states may influence arterial compliance, vascular tone, endothelial function, and wave reflection properties, all of which contribute to modifications in pulse waveform morphology and harmonic composition.

Although the translational applicability of these findings remains preliminary, radial pulse harmonic analysis may provide complementary physiological information beyond conventional biochemical or hemodynamic markers. Importantly, current evidence remains exploratory, and substantial multicenter validation, standardized acquisition protocols, and longitudinal investigations are still required before clinical implementation can be considered (7, 11). From a computational perspective, frequency-domain analysis offers several potential advantages for systemic physiological assessment, including non-invasive acquisition, relatively low computational complexity, and compatibility with wearable or photoplethysmography-based monitoring systems. Future integration with multimodal cardiovascular biomarkers, artificial intelligence-assisted analysis, and patient-specific hemodynamic modeling may further enhance the translational value of harmonic analysis in precision medicine applications.

Overall, current evidence supports the concept that radial pulse harmonic analysis may serve as a promising computational framework for investigating systemic vascular physiology; however, further validation is necessary before its broader clinical applicability can be established.

### 3.2 Hypertension

Hypertension is characterised by elevated systemic arterial pressure, increased arterial stiffness, and augmented wave reflection, all of which directly influence the spectral composition of arterial pulse waveforms (28, 29).

#### 3.2.1 Harmonic spectral shifts in hypertension

In patients with hypertension, a pronounced redistribution of harmonic energy toward higher-order harmonics has been repeatedly observed (42–44). The relative contribution of the fundamental harmonic (C_1_) is often reduced, whereas higher-order components (C_3_–C_7_) are significantly enhanced (40). This pattern reflects accelerated wave propagation and earlier wave reflection caused by elevated arterial stiffness.

Spectral centroid analyses further demonstrate a rightward shift of energy toward higher frequencies in hypertensive patients, indicating dominance of faster pressure oscillations (43). These alterations scale with hypertension severity and duration, suggesting that harmonic indices may serve as continuous markers of vascular load rather than binary disease indicators (44).

#### 3.2.2 Enhancement of high-order harmonics

High-order harmonics are particularly sensitive to changes in peripheral resistance and microvascular impedance (45). The elevation of these components is attributed to increased impedance mismatch at resistance arterioles and enhanced reflection coefficients.

Pharmacological intervention studies further demonstrate that after antihypertensive treatment, especially with agents improving arterial compliance, partial normalization of high-order harmonic amplitudes occurs in parallel with reductions in central systolic pressure and pulse pressure (43, 44).

#### 3.2.3 Association with central blood pressure and PWV

Strong correlations have been reported between harmonic indices and established hemodynamic markers of arterial stiffness. Relative high-order harmonic power correlates positively with carotid–femoral PWV, AIx, and central systolic blood pressure, whereas the fundamental harmonic shows inverse relationships (44, 45).

Regression models combining harmonic parameters and demographic variables show predictive accuracy for central blood pressure comparable to that of PWV alone (46), indicating that radial pulse harmonics capture key aspects of arterial stiffness and pressure amplification.

### 3.3 Diabetes mellitus

Diabetes mellitus is associated with microvascular dysfunction, endothelial damage, autonomic neuropathy, and accelerated arterial stiffening (13, 30), all of which profoundly affect harmonic characteristics of the radial pulse.

#### 3.3.1 Impact of microvascular dysfunction on harmonic energy

Patients with T2DM consistently show reduced low-frequency harmonics (C_1_–C_2_) and disproportionate elevation of mid- to high-frequency components (C_4_–C_7_) compared with non-diabetic controls (10). This pattern reflects impaired pulsatile energy transmission caused by increased microvascular resistance and reduced capillary compliance.

Longitudinal studies further demonstrate that worsening glycaemic control and duration of diabetes are associated with progressive harmonic distortion (47, 48), supporting harmonic indices as sensitive biomarkers of cumulative microvascular injury.

#### 3.3.2 Autonomic neuropathy and harmonic asymmetry

Autonomic dysfunction in diabetes manifests as impaired baroreflex sensitivity and dysregulated vascular tone (32, 49). Importantly, harmonic asymmetry becomes evident in early-stage diabetes before overt macrovascular complications appear (48, 50), supporting its role as an early functional marker of diabetic autonomic impairment.

In these five studies, non-invasive radial pulse wave recordings from patients with type 2 diabetes mellitus, applying harmonic analytic techniques such as Fourier transformation to decompose the waveform into frequency components, were utilized. They focus particularly on the amplitude variation of the fourth harmonic and the power of the first harmonic as key parameters. The study populations included diabetic cohorts monitored longitudinally for cardiovascular outcomes. Statistically significant associations were observed between altered harmonic indices and increased risks of macrovascular and microvascular events, silent myocardial ischemia, and major adverse cardiovascular events, with predictive values demonstrated through hazard ratios and receiver operating characteristic curves. These findings suggest that radial pulse harmonic features can serve as sensitive and quantitative biomarkers to improve cardiovascular risk stratification and early intervention strategies in diabetic populations (47–51).

### 3.4 Coronary artery disease

Coronary artery disease (CAD) is characterized by progressive atherosclerotic obstruction, diminished arterial compliance, and impaired ventricular–arterial coupling, all of which considerably modify the hemodynamic profile of arterial pressure waves (51).

Patients with angiographically confirmed CAD consistently exhibit steeper harmonic decay rates compared to healthy individuals (52). This pattern, marked by accelerated attenuation of low-order harmonics and a relative elevation of higher-order harmonic components, indicates increased arterial impedance and the premature return of reflected waves (53). Importantly, multivessel CAD demonstrates striking harmonic distortion compared to single-vessel disease, suggesting that harmonic indices are sensitive not only to the presence of CAD but also to the severity and extent of atherosclerotic involvement (54).

### 3.5 Colectomy in patients with colorectal cancer

Chuang et al. (54) reported significant changes in radial pulse parameters following colectomy in patients with colorectal cancer. Harmonic analysis revealed altered amplitude distributions in specific frequency components, with notable increases or decreases in the power of certain harmonics post-surgery. Time-domain pulse morphology indices also showed statistically significant shifts, indicating modifications in vascular compliance and autonomic regulation associated with the surgical intervention. These changes highlight the capacity of radial pulse wave analysis to capture physiological alterations related to colectomy, supporting its relevance in integrating traditional Chinese medicine diagnostics with objective cardiovascular monitoring.

### 3.6 Age-related migration of harmonic structure

Vascular aging involves progressive endothelial dysfunction, elastin degradation, collagen accumulation, and medial thickening, producing increased arterial stiffness and altered wave reflection (13, 30). Young adults exhibit dominant low-order harmonics with gradual decay at higher orders, whereas older individuals demonstrate marked reduction in low-frequency power and relative high-frequency enhancement (20). This rightward spectral migration reflects accelerated PWV and earlier reflected-wave return.

#### 3.6.1 Comparison with AIx and PWV

Harmonic indices correlate strongly with AIx and PWV but provide complementary physiological information (30). While PWV reflects global stiffness, harmonic parameters simultaneously encode ventricular ejection, peripheral resistance, and wave reflection effects (40, 55).

#### 3.6.2 Implications for precision vascular phenotyping

Distinct harmonic redistribution patterns have been reported across healthy aging, arterial stiffening, hypertension, diabetes mellitus, coronary artery disease, and other vascular-related physiological conditions (**Table 1**). These findings support the concept that radial pulse harmonic analysis may serve as a promising non-invasive framework for precision vascular phenotyping and computational cardiovascular assessment.

Through Fourier-based decomposition of RPP waveforms, harmonic analysis provides quantifiable frequency-domain biomarkers reflecting the complex interactions among cardiac ejection, arterial compliance, wave reflection, peripheral resistance, and autonomic regulation. Previous studies have demonstrated that specific harmonic alterations are associated with vascular remodeling, endothelial dysfunction, autonomic dysregulation, and age-related arterial stiffening. Importantly, these physiological changes may not always be fully captured by conventional blood pressure measurements or routine biochemical markers alone.

Taken together, current evidence suggests that radial pulse harmonic analysis represents a promising computational tool for non-invasive vascular assessment, early detection of physiological alterations, and longitudinal cardiovascular monitoring. Nevertheless, further multicenter validation, methodological standardization, and large-scale prospective studies remain necessary before broader clinical implementation can be fully established (**Table 1**).

### 3.7 Integration with emerging hemodynamic biomarkers

Recent advances in computational cardiovascular physiology have expanded the range of non-invasive hemodynamic biomarkers available for vascular assessment. In addition to harmonic analysis, Reservoir-excess pressure analysis (56–58) and photoplethysmography-derived indices (59, 60) have demonstrated translational potential in evaluating cerebrovascular health, renal dysfunction, vascular aging, and cardiovascular risk stratification.

Reservoir-excess pressure analysis provides complementary information regarding arterial reservoir function, wave propagation, and vascular compliance, while photoplethysmography-derived biomarkers offer scalable approaches for wearable cardiovascular monitoring. The integration of these multimodal physiological biomarkers with harmonic analysis may improve the sensitivity and specificity of non-invasive cardiometabolic assessment.

Future developments combining harmonic analysis with artificial intelligence-assisted interpretation, wearable sensing systems, and multimodal vascular biomarkers may facilitate personalized cardiovascular monitoring and precision medicine applications.

## 4 Discussion

In this systematic review, we synthesised the latest evidence on harmonic analysis of radial and arterial pulse waves, emphasizing its physiological underpinnings, technological evolution, and clinical applications across aging, metabolic disorders, cardiovascular diseases, and emerging digital-health platforms (3, 4, 8, 40). Collectively, the findings of the reviewed studies demonstrate that pulse waveform harmonic analysis provides a quantitative window into arterial stiffness, wave reflection, vascular compliance, and ventricular–arterial coupling, thereby complementing established hemodynamic indices such as PWV, augmentation index (AIx), and central blood pressure (5–7, 26, 28–30, 52).

From a computational biology and medicine perspective, traditional pulse diagnosis primarily serves as a historical and conceptual framework, whereas harmonic analysis offers a quantitative, reproducible, and model-based representation of vascular dynamics (1, 3, 7, 23, 40). This distinction is critical for positioning pulse harmonic analysis within contemporary biomedical engineering, computational physiology, and data-driven cardiovascular assessment (4, 9, 27, 61).

Despite substantial methodological and clinical progress over the past four decades (**Table 1**, **Figure 2**), challenges remain in standardization, mechanistic interpretation, and large-scale clinical validation, which must be addressed before harmonic analysis can be fully translated into routine clinical decision-making (6, 15, 25, 39).

**Table 1:** Representative clinical and translational applications of radial pulse harmonic analysis and emerging computational cardiovascular approaches.

Clinical condition/physiological state	Representative harmonic alterations	Physiological interpretation	Translational/clinical relevance	Representative references
Hypertension	Increased higher-order harmonics; altered harmonic redistribution	Increased arterial stiffness, wave reflection, and vascular resistance	Assessment of vascular remodeling and hemodynamic burden	(11, 19, 28–30, 40, 43–45)
Type 2 diabetes mellitus	Reduced low-order harmonic components; impaired harmonic variability	Autonomic dysfunction, endothelial impairment, and microvascular dysregulation	Early cardiometabolic risk assessment and autonomic evaluation	(10, 13, 32, 47–49, 51)
Coronary artery disease	Altered harmonic spectral distribution and pulse waveform morphology	Impaired arterial compliance and abnormal coronary hemodynamics	Non-invasive cardiovascular risk stratification	(51–54)
Vascular aging	Harmonic redistribution with reduced low-frequency dominance	Progressive arterial stiffening and vascular remodeling	Evaluation of age-related vascular dysfunction	(13, 20, 30),
Autonomic dysfunction	Reduced harmonic complexity and altered frequency-domain balance	Impaired autonomic regulation and baroreflex dysfunction	Physiological monitoring of autonomic cardiovascular control	(35–38)
Inflammatory and systemic physiological conditions	Exploratory harmonic alterations associated with vascular modulation	Endothelial dysfunction, inflammatory vascular response, and altered vascular tone	Exploratory systemic physiological assessment	(39–42)

PPG, photoplethysmography.

Some studies contributed to multiple clinical or methodological application domains and are therefore listed in more than one category within the table (e.g., references (30, 40) and (51)).

### 4.1 Conceptual framework and clinical implications of radial pulse harmonic analysis

The arterial pressure waveform contains rich frequency-domain information reflecting vascular compliance, peripheral resistance, impedance mismatch, and the interaction between forward and reflected pressure waves (26, 27, 33, 36). The conceptual foundation of harmonic analysis lies in Fourier-based decomposition, in which complex pulsatile signals are expressed as a finite sum of sinusoidal components with distinct amplitudes and phases (8, 32). Experimental and theoretical studies consistently show that arterial stiffening leads to systematic redistribution of harmonic energy, characterized by attenuation of low-order harmonics and relative amplification or distortion of higher-order components (9, 11, 14, 34). Transmission-line and impedance-based arterial models further support this interpretation, demonstrating that reduced arterial compliance and increased characteristic impedance alter both amplitude and phase relationships across harmonics (11, 28, 33, 38). Importantly, emerging mechanobiological evidence demonstrates that endothelial cells respond selectively to frequency-specific hemodynamic oscillations. Specific harmonic components modulate inflammatory signaling, oxidative stress pathways, and endothelial phenotype (44), providing a mechanistic bridge between frequency-domain pulse analysis and vascular biology (20, 42).

As illustrated in **Figure 2**, radial pulse harmonic analysis provides a structured framework for transforming non-invasive arterial pulse signals into quantitative and physiologically meaningful biomarkers. Through standardized signal acquisition, preprocessing, and peak alignment, variability in RPP signals can be effectively reduced, thereby enabling consistent characterization of pulse wave dynamics across individuals (7, 18, 38). Such methodological standardization is essential for improving reproducibility and facilitating clinical translation (6, 25). By applying Fourier-based harmonic decomposition, complex pulse waveforms can be represented in the frequency domain, where individual harmonic components reflect distinct aspects of cardiovascular function (8, 32). In general, lower-order harmonics are associated with cardiac output and central arterial compliance, whereas higher-order harmonics are more sensitive to peripheral resistance, wave reflection, and microvascular function (10, 11, 19, 32, 33). This frequency-domain approach provides a mechanistic perspective that complements conventional time-domain indices and aligns with established models of arterial hemodynamics (26–28, 33). These results highlight the sensitivity of harmonic analysis to early systemic physiological disturbances that may not be captured by routine clinical assessments. Consistent with previous reports, hypertension was associated with a redistribution of spectral energy toward higher-order harmonics, reflecting increased arterial stiffness and enhanced wave reflection (40, 43, 44, 61). These alterations are in agreement with established hemodynamic changes, including elevated vascular impedance and earlier return of reflected waves, and are closely related to conventional markers such as pulse wave velocity and central blood pressure (28, 44, 45). Similarly, in diabetes mellitus, reductions in low-order harmonics (C_1_–C_2_) and relative increases in mid- to high-order components (C_4_–C_7_) have been reported, indicating the presence of microvascular dysfunction and autonomic neuropathy (10, 47–50). These patterns suggest that harmonic indices are capable of capturing both macrovascular and microvascular alterations, providing a more comprehensive assessment of vascular health.

Overall, the framework presented in **Figure 2** emphasizes the translational potential of radial pulse harmonic analysis as a non-invasive and physiologically interpretable tool for cardiovascular and metabolic assessment. By integrating signal processing with clinical interpretation, harmonic analysis may contribute to early disease detection, improved risk stratification, and the development of precision medicine approaches, particularly when combined with emerging digital health technologies and data-driven methodologies (14, 24, 46).

### 4.2 Advances in pulse wave acquisition and digital technologies

Reliable harmonic analysis fundamentally depends on high-quality pulse waveform acquisition. Traditional subjective palpation methods have been progressively replaced or augmented by objective sensor-based systems, including applanation tonometry, strain-gauge sensors, piezoelectric transducers, and PPG (2, 7, 12, 15, 17).

Standardized acquisition protocols, particularly those defining sensor positioning, applied contact pressure, sampling resolution, and signal preprocessing, have markedly improved reproducibility and inter-study comparability (7, 15, 25). Such standardization is essential to ensure consistency across devices, populations, and computational pipelines (6, 39).

PPG-based harmonic extraction has attracted increasing attention due to its non-invasiveness, scalability, and compatibility with wearable and remote monitoring systems (17, 18, 21, 24). Recent validation studies confirm that frequency-domain PPG features correlate strongly with vascular load, systolic blood pressure, and arterial stiffness (19), positioning harmonic analysis as a promising feature set for digital health platforms (21, 24).

Furthermore, machine learning approaches have expanded the analytical capabilities of harmonic indices. Harmonic features have been integrated into predictive models for estimating arterial compliance, characteristic impedance, and cardiometabolic risk (14, 24, 46), aligning with the Frontiers emphasis on computational modelling and translational applicability (4, 61).

### 4.3 Clinical applications across disease domains

#### 4.3.1 Aging and arterial stiffness

Vascular aging involves progressive arterial stiffening, endothelial dysfunction, and altered pulse wave morphology (13, 20, 26). Harmonic indices shift systematically with age, showing increased variability and phase alteration in higher-order harmonics (3, 8, 32, 40). These changes parallel established increases in PWV and reductions in dynamic arterial compliance (5, 13, 35).

Harmonic-based parameters demonstrate predictive value for cardiovascular risk comparable to traditional stiffness indices (5, 46, 52), suggesting utility for early detection and longitudinal assessment of vascular aging (4, 62).

#### 4.3.2 Diabetes and metabolic disorders

Type 2 diabetes mellitus induces widespread vascular dysfunction mediated by endothelial impairment, autonomic neuropathy, oxidative stress, and accelerated arterial stiffening (35, 42, 51). Harmonic analysis reveals altered amplitude ratios, particularly involving the third and fourth harmonics, reflecting increased peripheral resistance and impaired damping capacity (10, 14, 31).

Longitudinal and outcome-based studies further demonstrate that variations in specific harmonic components predict future macrovascular and microvascular events (47–50), supporting harmonic indices as sensitive computational biomarkers of diabetic vascular dysfunction (49, 51).

#### 4.3.3 Coronary artery disease and atherosclerosis

Coronary artery disease alters arterial impedance and wave reflection patterns, producing distinctive harmonic signatures (40, 41, 53). Patients with angiographically confirmed CAD exhibit abnormal harmonic decay and increased spectral distortion, consistent with stiffened conduit arteries and disrupted flow dynamics (50, 53).

Evidence suggests that harmonic parameters reflect both the presence and severity of CAD, including multivessel involvement (50, 53). Integration with central blood pressure estimation and stress testing further enhances diagnostic performance (29, 37, 41).

#### 4.3.4 Hypertension and cardiovascular remodeling

Hypertension is characterized by progressive alterations in arterial compliance, vascular impedance, wave reflection, and ventricular–arterial coupling, ultimately leading to structural and functional remodeling of the cardiovascular system (20, 52). These hemodynamic changes contribute to increased arterial stiffness and altered pulse wave propagation, which can be quantitatively captured through harmonic analysis of radial pulse waveforms.

Previous studies have demonstrated that hypertensive individuals exhibit characteristic changes in harmonic energy distribution, including increased contributions from higher-order harmonic components and reduced harmonic damping (9, 11, 34). These spectral alterations are thought to reflect enhanced wave reflection, elevated vascular resistance, and impaired arterial elasticity. Importantly, such harmonic signatures are consistent with established hemodynamic markers, including pulse wave velocity, arterial input impedance, and measures of vascular stiffness (28, 38, 39).

From a translational perspective, harmonic analysis provides a non-invasive and physiologically interpretable approach for assessing vascular remodeling associated with hypertension. The ability to detect subtle alterations in pulse waveform characteristics may offer complementary information beyond conventional blood pressure measurements and support early identification of vascular dysfunction, cardiovascular risk stratification, and therapeutic monitoring (52, 56).

### 4.4 Integration with traditional pulse diagnosis

Traditional pulse diagnosis has historically relied on qualitative tactile descriptors (16). Harmonic analysis provides an objective, mathematical reinterpretation of pulse characteristics, transforming subjective impressions into quantifiable spectral features (7, 12, 23).

From an engineering viewpoint, traditional concepts serve mainly as a historical and hypothesis-generating framework rather than a diagnostic endpoint (1, 3, 15, 40). Harmonic analysis, grounded in signal processing and vascular mechanics, offers reproducible biomarkers aligned with modern biomedical engineering (4, 61).

### 4.5 Methodological challenges and standardization needs

Despite the growing body of evidence supporting the clinical and physiological relevance of radial pulse harmonic analysis, several important challenges continue to limit its broader translational adoption. First, substantial variability exists among pulse acquisition systems, including differences in sensor technologies, measurement configurations, sampling frequencies, and signal quality, which may influence the reproducibility and comparability of harmonic indices across studies (15, 17). Second, the absence of standardized preprocessing and harmonic computation pipelines, including procedures for baseline correction, peak alignment, ensemble averaging, normalization, and harmonic extraction, remains a major obstacle to cross-study validation and clinical implementation (6, 25, 32).

In addition, harmonic parameters may be influenced by multiple physiological confounders, including age, sex, autonomic activity, blood pressure status, vascular stiffness, and comorbid cardiovascular or metabolic conditions (26, 36, 39). These factors may complicate the interpretation of harmonic patterns and contribute to inter-individual variability. Furthermore, although lower-order harmonic components have been linked to specific hemodynamic mechanisms, the physiological interpretation and clinical significance of higher-order harmonics remain incompletely understood and require further mechanistic investigation (11, 44).

Finally, most existing studies have been conducted in relatively small or single-center cohorts, limiting the generalizability of current findings. Large-scale multicenter studies, standardized methodological frameworks, and prospective validation across diverse populations will be essential to establish robust reference standards and facilitate the integration of harmonic analysis into routine cardiovascular assessment and precision medicine applications (3, 41).

### 4.6 Future directions and overall interpretation

Recent advances in computational cardiovascular physiology have expanded the range of non-invasive hemodynamic biomarkers available for vascular assessment beyond conventional blood pressure measurements. In addition to radial pulse harmonic analysis, reservoir-excess pressure analysis has emerged (56–58) as a promising approach for characterizing vascular compliance, wave reflection dynamics, and arterial reservoir function. Previous studies have demonstrated significant associations between Reservoir-Excess Pressure parameters and cerebrovascular health, renal dysfunction, and cardiovascular risk stratification (56–58). These findings suggest that advanced hemodynamic waveform analysis may provide complementary physiological information regarding systemic vascular remodeling and target-organ damage.

Similarly, photoplethysmography (PPG)-derived indices have gained increasing attention as scalable and non-invasive biomarkers for cardiovascular monitoring, particularly in wearable and digital health applications (59). The integration of frequency-domain pulse waveform analysis with wearable sensing technologies may facilitate continuous and real-time assessment of vascular physiology in both clinical and community settings. Nevertheless, previous investigations have suggested that comorbid conditions may attenuate the diagnostic performance and physiological specificity of PPG-derived biomarkers (59, 60). Therefore, future translational studies should emphasize multimodal physiological integration, standardized acquisition protocols, and large-scale validation across diverse patient populations.

Taken together, these emerging approaches support the concept that radial pulse harmonic analysis should be interpreted within the broader framework of computational hemodynamics and multimodal cardiovascular biomarker assessment. Future integration with Reservoir-Excess Pressure Analysis, photoplethysmography-derived biomarkers, artificial intelligence-assisted interpretation, patient-specific computational modeling, and computational fluid dynamics (CFD) (63, 64) may further enhance the translational value of non-invasive vascular assessment in precision cardiovascular medicine. In particular, CFD-based approaches may provide additional mechanistic insights into three-dimensional vascular flow distribution, wave propagation, and peripheral circulatory dynamics, thereby complementing conventional pulse waveform analysis and advancing the physiological interpretation of vascular hemodynamics (63, 64).

An additional area that may warrant future investigation is the potential application of pulse waveform harmonic analysis in systemic disorders beyond traditional cardiovascular and metabolic diseases. Because harmonic parameters reflect integrated interactions among vascular function, autonomic regulation, and systemic hemodynamics, it is conceivable that certain non-cardiovascular conditions associated with inflammation, metabolic dysregulation, or endothelial dysfunction may also influence harmonic patterns. Preliminary observations (65) from small exploratory studies have suggested that viral hepatitis and other chronic systemic disorders may be associated with subtle alterations in pulse waveform characteristics. However, the physiological mechanisms underlying these observations remain poorly understood, and their clinical significance has yet to be established. Further mechanistic and prospective studies are therefore required before any potential diagnostic or screening applications can be considered. In conclusion, harmonic analysis of arterial pulse waves represents a robust and physiologically grounded computational framework for vascular assessment **(Table 1**). Although challenges remain, advances in signal acquisition, modelling, and artificial intelligence position harmonic analysis as a promising tool for cardiovascular risk assessment and digital health translation (3, 4, 61, 62).

## 5 Conclusion

This Mini Review comprehensively examined the physiological foundations, methodological developments, and clinical applications of radial pulse harmonic analysis as a frequency-domain framework for non-invasive cardiovascular assessment. The accumulated evidence indicates that harmonic analysis provides quantitative and physiologically interpretable information regarding arterial compliance, wave reflection, vascular resistance, arterial stiffness, and ventricular–arterial coupling, thereby offering valuable insights into systemic hemodynamics and vascular function. From a biomedical engineering perspective, harmonic decomposition transforms complex radial pressure pulse waveforms into structured frequency-domain biomarkers that can be objectively quantified, computationally analyzed, and compared across populations. By capturing spectral characteristics associated with vascular remodeling, endothelial dysfunction, autonomic regulation, and age-related arterial changes, harmonic parameters may complement established cardiovascular indices such as pulse wave velocity, augmentation index, central blood pressure, and other hemodynamic biomarkers.

The reviewed literature demonstrates that radial pulse harmonic analysis has potential applications in hypertension, diabetes mellitus, coronary artery disease, vascular aging, and autonomic dysfunction. Moreover, recent advances in sensor technology, signal acquisition systems, wearable devices, photoplethysmography-derived biomarkers, Reservoir-Excess Pressure Analysis, and computational cardiovascular modeling have expanded the translational potential of pulse waveform analysis. These developments support the emerging concept of multimodal cardiovascular assessment, in which harmonic analysis may serve as one component of a broader physiological biomarker framework. Despite these promising advances, several challenges remain, including methodological heterogeneity, lack of standardized acquisition and preprocessing protocols, incomplete physiological understanding of certain harmonic components, and limited multicenter validation. Addressing these challenges will be essential for improving reproducibility, facilitating cross-study comparisons, and establishing clinically meaningful reference standards.

Future research should focus on large-scale prospective validation, integration with multimodal physiological sensing technologies, artificial intelligence-assisted interpretation, patient-specific computational modeling, and computational fluid dynamics-based hemodynamic assessment. Such efforts may further enhance the physiological interpretation, clinical applicability, and translational value of harmonic analysis within the evolving field of precision cardiovascular medicine.

In conclusion, radial pulse harmonic analysis represents a promising computational and translational approach for non-invasive vascular assessment. With continued methodological standardization, mechanistic validation, and technological advancement, it has considerable potential to contribute to early cardiovascular risk detection, longitudinal physiological monitoring, and precision cardiometabolic healthcare.
